# Amyotrophic lateral sclerosis (ALS) among immigrant groups and Swedish-born individuals: a cohort study of all adults 18 years of age and older in Sweden

**DOI:** 10.1007/s00415-021-10765-6

**Published:** 2021-08-24

**Authors:** Per Wändell, Sten Fredrikson, Axel C. Carlsson, Xinjun Li, Jan Sundquist, Kristina Sundquist

**Affiliations:** 1 Division of Family Medicine and Primary Care, Department of Neurobiology, Care Sciences and Society, Karolinska Institutet, Alfred Nobels Allé 23, 141 83 Huddinge, Sweden; 2 Division of Neurology, Department of Clinical Neuroscience, Karolinska Institutet Huddinge, Stockholm, Sweden; 3 Academic Primary Health Care Centre, Stockholm Region, Stockholm, Sweden; 4 Center for Primary Health Care Research, Lund University, Malmö, Sweden; 5 Department of Family Medicine and Community Health, Department of Population Health Science and Policy, Icahn School of Medicine At Mount Sinai, New York, NY USA; 6 Center for Community-Based Healthcare Research and Education (CoHRE), Department of Functional Pathology, School of Medicine, Matsue, Shimane University, Matsue, Japan

**Keywords:** ALS, Gender, Immigrants, Neighborhood, Socioeconomic status

## Abstract

**Background:**

There is a lack of studies of amyotrophic lateral sclerosis (ALS) in immigrants.

**Objective:**

The objective is to study the association between country of birth and incident ALS in first-generation immigrants versus Swedish-born individuals, and in second-generation immigrants versus native Swedes.

**Methods:**

Study populations included all adults aged 18 years and older in Sweden, in the first-generation study 6,128,698 individuals (2,975,141 men, 3,153,557 women) with 5,344 ALS cases (3017 men, 2327 women), and in the second-generation study 4,588,845 individuals (2,346,855 men and 2,241,990 women) with 3,420 cases (2027 men and 1393 women). ALS was defined as having at least one registered diagnosis of ALS in the National Patient Register 1998–2017. The incidence of ALS in different first-generation immigrant groups versus Swedish-born individuals was assessed by Cox regression, expressed as hazard ratios (HRs) with 95% confidence intervals (CI). The models were stratified by sex and adjusted for age, geographical residence in Sweden, educational level, marital status, and neighbourhood socioeconomic status.

**Results:**

After adjusting for potential confounders, the HRs were lower in foreign-born men, 0.71 (95% CI 0.63–0.81), and women, 0.80 (95% CI 0.70–0.92). The ALS risk was lower among men and women from most Western countries (Europe outside Nordic countries, and North America), and from other regions of the world (Africa, Asia, and Latin America). Among men and women with foreign-born parents, the risk of ALS did not differ significantly from native Swedes.

**Significance:**

In general, the risk of ALS was lower in first-generation men and women but did not differ in second-generation individuals.

**Supplementary Information:**

The online version contains supplementary material available at 10.1007/s00415-021-10765-6.

## Introduction

Amyotrophic lateral sclerosis (ALS), Lou Gehrig's disease, is a neurodegenerative disease of motor neurons, both in primary motor cortex, cortico-spinal tracts, brainstem and spinal cord [1]. The disease is characterized by a progressive muscular paralysis that does not affect the sensory neurons, the senses or mental functioning [2].

The prevalence of ALS is rather uniform in Western countries, with 5.2 per 100,000 [1]. The mean onset of ALS is estimated at 60 years of age for sporadic cases [1], with a slight male preponderance, M/F ratio around 1.5. The disease leads to death in respiratory failure usually within 2–5 years, earlier in the bulbar onset type (primary bulbar palsy, PBP) and later in the spinal onset type (classical or Charcot’s ALS) [2, 3].

The incidence of ALS in the world in recent studies is estimated to between 0.6 and 3.8 per 100,000 person-years [4], with an estimated pooled incidence of 1.7 per 100,000 person-years [5]. However, the incidence differs throughout the world, with a standardized incidence rate in Northern Europe of 1.89 per 100,000 person-years, compared to East Asia with 0.83 per 100,000 person-years, and South Asia with 0.73 per 100,000 person-years [5]. In three European countries, i.e. Italy, Ireland and the UK, the incidence has been estimated between 2.2 per 100,000 [3], but an increasing incidence has been shown with up to 3.8 per 100,000 in Scotland [6]. In Sweden, the incidence is estimated at 3.8 per 100,000 person-years, using data from a population-based motor neuron disease register [7].

These differences by region of origin led us to our aim in the present study, i.e., to estimate the incidence of ALS in first- and second-generation immigrants in Sweden compared to Swedish-born individuals and Swedish-born individuals with Swedish-born parents, respectively.

## Methods

### Design

The nationwide registers used in the present study were the Total Population Register, including data on socio-economic factors and country of birth, and the National Patient Register (NPR), including diagnoses from hospital care and from 2001 also from out-patient care from hospitals. Subjects aged 18 years of age and older were included in the study. The follow-up period ran from January 1, 1998 until hospitalisation/out-patient diagnosis of ALS at age 18 years or more, death, emigration or the end of the study period on December 31, 2017, whichever came first. Out-patient diagnoses were included nationwide from 2001 and onwards from specialist care but not primary health care. First- and second-generation immigrants were included, divided by region of origin or by region of origin in the parents of the individuals, respectively. This study was approved by the Regional Ethical Review Board in Lund, Sweden (No. 2012/795). Participant consent was not required as this study used only pseudonymized registry-based administrative secondary data.

### Outcome variable

ALS (Amyotrophic Lateral Sclerosis) or Familial Motor Neuron Disease (G12.2).

### Co-morbidities

We also identified co-morbidities according to ICD-10 for the following diagnoses: diabetes (E10-E14), heart diseases (I05-09, I11, I13, I20-25, I30-43, I48, I50, I51-53) and dementia (F00, F01, F02, F03, G30, and G31.8A).

### Demographic and socioeconomic variables

The study population was stratified by *sex*.

*Age* was used as a continuous variable in the analysis.

*Educational attainment* was categorised as ≤ 9 years (partial or complete compulsory schooling), 10–12 years (partial or complete secondary schooling) and > 12 years (attendance at college and/or university).

*Marital status* was categorized as married or not being married.

*Geographic region of residence* was included in order to adjust for possible regional differences in hospital admissions and was categorised as (1) large cities, (2) southern Sweden and (3) northern Sweden. Large cities were defined as municipalities with a population of > 200,000 and comprised the three largest cities in Sweden: Stockholm, Gothenburg and Malmö.

*Region of origin* of first-generation immigrants, or of the parents to second-generation immigrants, were categorized into 1. Nordic countries, 2. Europe (with the exclusion of Nordic countries) and North America, and 3. Asia, Africa and Latin America.

### Neighbourhood deprivation

The neighborhood deprivation index was categorized into four groups: more than one standard deviation (SD) below the mean (low deprivation level or high socio-economic status (SES)), more than one SD above the mean (high deprivation level or low SES), and within one SD of the mean (moderate SES or moderate deprivation level) used as reference group, and also unknown neighborhood SES.

### Statistical analysis

Continuous variables are presented as mean and standard deviations, and categorical variables are presented as counts and percentages. Cox regression analysis was used for estimating the risk (hazard ratios (HR) with 95% confidence intervals (CI)) of incident ALS in different immigrant groups compared to the Swedish-born population during the follow-up time. For the second-generation immigrants, Swedish-born individuals with Swedish-born parents were used as referents. All analyses were stratified by sex. Three models were used in our analyses: Model 1 was adjusted for age and region of residence in Sweden, and Model 2 for age, region of residence in Sweden, educational level, marital status and neighborhood SES, and Model 3 as Model 3 but also for co-morbidities (diabetes, heart diseases and dementia). We also performed an age-stratified analysis in the first-generation study, i.e., in the age intervals 18–59 years of age, and ≥ 60 years of age.

## Results

In the first-generation study (Table 1, Supplementary Tables 1a, b), totally 6,128,698 individuals (2,975,141 men and 3,153,557 women) with 5,344 ALS cases (3017 men and 2327 women). For the first-generation study, the age-standardised incidence in the ages 18 years and above was for men and women, respectively, 6.6 and 4.6 per 100,000 patient-years (Supplementary Fig. S1); and for all Swedish-born and foreign-born, respectively, 5.9 and 3.4 per 100,000 patient-years (Supplementary Fig. S2), with the peak incidence rate in the years 75–79 years of age. In the second-generation study (Table 1, Supplementary Tables 2a, b), 4,588,845 individuals (2,346,855 men and 2,241,990 women) with 3,420 cases (2027 men and 1393 women). For the second-generation study, the age-standardised incidence in the ages 18 years and above was for men and women, respectively, 4.7 and 3.3 per 100,000 patient-years (Supplementary Fig. S3); and for native Swedes and individuals with foreign-born parents, respectively, 4.4 and 2.1 per 100,000 patient years (Supplementary Fig. S4), with the peak incidence rate for all men and women and all native Swedes 80 years and above, and for individuals with foreign-born parents in the years 75–79 years of age.

**Table 1:** Characteristics of the study populations in first-generation and the second-generation studies and the number of cases of ALS in each group

	First-generation individuals				Second-generation individuals			
	Population		Cases		Population		Cases	
	No.	%	No.	%	No.	%	No.	%
Total population	6,128,698		5344		4,588,845		3420	
Gender								
Males	2,975,141	48.5	3017	56.5	2,346,855	51.1	2027	59.3
Females	3,153,557	51.5	2327	43.5	2,241,990	48.9	1393	40.7
Immigrant status*								
Swedish	5,093,177	83.1	4800	89.8	4,056,228	88.4	3163	92.5
Foreign born	1,035,521	16.9	544	10.2	532,617	11.6	257	7.5
Age (years)								
18–39	2,337,436	38.1	362	6.8	2,278,761	49.7	394	11.5
40–49	1,042,606	17.0	726	13.6	989,623	21.6	746	21.8
50–59	1,067,667	17.4	1539	28.8	957,788	20.9	1455	42.5
60 +	1,680,989	27.4	2717	50.8	362,673	7.9	825	24.1
Educational level								
≤ 9	2,291,344	37.4	2209	41.3	1,125,014	24.5	1100	32.2
10–11	2,070,945	33.8	1880	35.2	2,169,870	47.3	1473	43.1
≥ 12	1,766,409	28.8	1255	23.5	1,293,961	28.2	847	24.8
Region of residence								
Large cities	2,713,033	44.3	2513	47.0	2,190,792	47.7	1605	46.9
Southern Sweden	1,908,984	31.1	1734	32.4	1,570,877	34.2	1108	32.4
Northern Sweden	1,506,681	24.6	1097	20.5	827,176	18.0	707	20.7
Marital status								
Married	3,478,186	56.8	3680	68.9	1,992,644	43.4	2123	62.1
Not married	2,650,512	43.2	1664	31.1	2,596,201	56.6	1297	37.9
Neighborhood deprivation								
Low	860,478	14.0	898	16.8	799,400	17.4	602	17.6
Middle	2,808,862	45.8	2599	48.6	2,514,367	54.8	1854	54.2
High	761,072	12.4	666	12.5	609,956	13.3	431	12.6
Unknown	1,698,286	27.7	1181	22.1	665,122	14.5	533	15.6
Hospital diagnosis of diabetes	404,462	6.6	386	7.2	242,666	5.3	230	6.7
Hospital diagnosis of Heart diseases	1,080,713	17.6	1063	19.9	481,120	10.5	512	15.0
Hospital diagnosis of dementia	174,119	2.8	184	3.4	35,882	0.8	112	3.3

*Immigration status in the second-generation was defined according to parental birth countries

In the first-generation study, among foreign-born men, the risk of ALS was higher for the two lower educational levels (Supplementary Table 3), and among foreign-born women, the risk of ALS was lower in the mid-level of education (Supplementary Table 4). For marital status, the ALS risk was higher among Swedish-born men and women in the first-generation study (Supplementary Tables 3 and 4). For region in Sweden, the risk was lower for Swedish-born men for both Southern and Northern Sweden, and for foreign-born men in Northern Sweden, while in the second-generation study, the risk of ALS was higher for native individuals in Northern Sweden (Supplementary Table 5). For neighborhood deprivation level, among foreign-born man and women the risk was lower among those living in neighbourhoods with high or unknown deprivation level, and for Swedish-born women the risk was borderline lower for those living in middle deprivation level.

As regards co-morbidities, the risk of ALS was lower for Swedish-born men and women for diabetes, heart diseases and dementia, and for foreign-born men and women for heart diseases; in the second-generation study the risk of ALS was lower for native Swedes for diabetes and heart diseases, but higher for dementia.

The male to female ratio was in the first-generation study was 1.37, and it was 1.39 in the second-generation. As regards age, in the first-generation study the mean and median age, respectively, was for Swedish-born men 67.9 years (sd 11.2) and 69 years, respectively, and for women 69.3 years (sd 11.5) and 70, respectively; and for foreign-born men 64.7 years (sd 11.2) and 66 years, respectively, and for foreign-born women 65.9 years (sd 12.0) and 67, respectively. In the second-generation study the corresponding mean and median ages were for men with Swedish-born parents 62.4 years (sd 9.9) and 63, and for women with Swedish-born parents 63.3 years (sd 9.9) and 65, and for men with foreign-born parents 56.5 years (sd 11.3) and 56, and for women with foreign-born parents 57.1 years (sd 10.4) and 58, respectively.

In the first-generation study (Table 2), among foreign-born men, the fully adjusted HR was 0.71 (95% CI 0.63–0.81), and among foreign-born women, the fully adjusted HR was 0.80 (95% CI 0.70–0.92). The risk of ALS was lower among men and women from Western countries (Europe outside Nordic countries, and North America), and from other regions of the world (Africa, Asia, and Latin America). In the age-stratified analysis the results were rather similar to those in the main analysis (Table 3).

**Table 2:** The relative risk of ALS in the first-generation immigrants expressed as hazard ratios (HR) with 95% confidence intervals (95% CI)

	(*n*)	Model 1			Model 2			Model 3		
		HR	95% CI		HR	95% CI		HR	95% CI	
Men										
Sweden	2716	1			1			1		
All foreign-born	301	**0.95**	**0.95**	**0.95**	**0.73**	**0.64**	**0.82**	**0.71**	**0.63**	**0.81**
Nordic countries	142	**0.80**	**0.68**	**0.95**	0.88	0.74	1.05	0.86	0.72	1.03
Western countries (rest of Europe, North America)	95	**0.60**	**0.49**	**0.73**	**0.62**	**0.50**	**0.76**	**0.61**	**0.49**	**0.74**
Others (Africa, Asia, and Latin America)	64	**0.60**	**0.47**	**0.77**	**0.64**	**0.49**	**0.82**	**0.65**	**0.50**	**0.83**
Women										
Sweden	2084	1			1			1		
All foreign-born	243	**0.96**	**0.95**	**0.96**	**0.81**	**0.70**	**0.93**	**0.80**	**0.70**	**0.92**
Nordic countries	158	1.07	0.91	1.26	1.07	0.91	1.26	1.06	0.89	1.25
Western countries (rest of Europe, North America)	61	**0.64**	**0.50**	**0.83**	**0.63**	**0.49**	**0.82**	**0.62**	**0.48**	**0.81**
Others (Africa, Asia, and Latin America)	24	**0.41**	**0.27**	**0.61**	**0.40**	**0.27**	**0.61**	**0.42**	**0.28**	**0.63**

Model 1: adjusted for age and region of residence in Sweden; model 2: adjusted for age, region of residence in Sweden, educational level, marital status, and neighborhood deprivations; model 3: model 2 + comorbidities

(*n*)  Observed numbers, *HR* hazard ratio, *CI* confidence interval

Bold values are statistically significant

**Table 3:** The age-stratified relative risk of ALS in first-generation immigrants expressed as hazard ratios (HR) with 95% confidence intervals (95% CI)

	Aged 18–59 years				Aged 60 + years			
	(*n*)	HR*	95% CI		(*n*)	HR*	95% CI	
Men								
Sweden	1352	1			1363	1		
All foreign-born	176	**0.72**	**0.61**	**0.85**	125	**0.64**	**0.53**	**0.77**
Nordic countries	73	0.80	0.63	1.02	69	**0.73**	**0.57**	**0.94**
Western Countries (including North America)	48	**0.57**	**0.43**	**0.76**	47	**0.57**	**0.43**	**0.77**
Others	55	0.81	0.62	1.07	9	**0.47**	**0.25**	**0.92**
Women								
Sweden	961	1			1122	1		
All foreign-born	137	0.87	0.72	1.05	106	**0.72**	**0.59**	**0.89**
Nordic countries	82	1.07	0.85	1.36	76	0.87	0.69	1.10
Western Countries (including North America)	34	0.71	0.50	1.01	27	**0.52**	**0.36**	**0.77**
Others	21	**0.62**	**0.40**	**0.96**	3	0.38	0.12	1.19

(*n*)  Observed numbers, *HR* hazard ratio, *CI* confidence interval

^*^Full adjusted

Bold values are statistically significant

In the second-generation study (Table 4), among men with foreign-born parents the fully adjusted HR was 1.10 (95% CI 0.93–1.31), and among women with foreign-born parents the fully adjusted HR was 1.17 (95% CI 0.96–1.43). The risk of ALS was borderline significant among women with parents from the Nordic countries.

**Table 4:** The relative risk of ALS in the second-generation immigrants expressed as hazard ratios (HR) with 95% confidence intervals (95% CI)

	(*n*)	Model 1			Model 2			Model 3		
		HR	95% CI		HR	95% CI		HR	95% CI	
Men										
Sweden	1877	1			1			1		
All with foreign-born parents	149	1.11	0.94	1.31	1.10	0.93	1.30	1.10	0.93	1.31
Nordic countries	93	1.15	0.93	1.42	1.13	0.92	1.40	1.14	0.92	1.40
Western Countries (rest of Europe, North America)	48	1.05	0.78	1.39	1.04	0.78	1.39	1.04	0.78	1.39
Others (Africa, Asia, and Latin America)	8	1.09	0.57	2.10	1.08	0.56	2.09	1.10	0.57	2.12
Women										
Sweden	1286	1			1			1		
All with foreign-born parents	106	1.19	0.97	1.45	1.17	0.96	1.43	1.17	0.96	1.43
Nordic countries	71	**1.30**	**1.02**	**1.66**	**1.27**	**1.00**	**1.62**	**1.28**	**1.00**	**1.63**
Western Countries (rest of Europe, North America)	34	1.13	0.80	1.59	1.12	0.79	1.58	1.12	0.80	1.58
Others (Africa, Asia, and Latin America)	1	0.38	0.09	1.51	0.36	0.09	1.44	0.36	0.09	1.46

Model 1: adjusted for age and region of residence in Sweden; model 2: adjusted for age, region of residence in Sweden, educational level, marital status, and neighborhood deprivations; model 3: model 2 + comorbidities

(*n*) Observed numbers, *HR* hazard ratio, *CI* confidence interval

Bold values are statistically significant

## Discussion

The main findings of this study were significantly lower risks of ALS among foreign-born men and women, but not among men and women born in Sweden with foreign-born parents. Among foreign-born men and women, the risk of ALS was lower among men and women from Europe outside Nordic countries, and from Asia, Africa or Latin America, while a borderline significantly higher risk was found among women with parents from the Nordic countries.

We choose to categorize first- and second-generation immigrants into groups. We could expect a similar risk in Western first-generation immigrants but found this only for men and women from the Nordic countries, while the risk was lower in men and women from the rest of Europe and North America. The “healthy migrant effect” is often mentioned as an explanation to a lower morbidity among foreign-born [8], and sometimes also the “salmon bias” or “re-migration bias” [9], i.e. that migrants tend to go back to their country of origin when expecting to die and possibly in this case when they contract symptoms of ALS. However, Danish studies found the “healthy migrant effect” questionable [10], and furthermore found no evidence for the “salmon bias” hypothesis to be true [11].

Regarding the third group, i.e. individuals from Asia, Africa or Latin America, there are previous studies showing a lower risk of ALS, especially among Asian populations [12, 13], i.e. in line with our findings. However, studies from Africa and Latin America are scarce, but with a low prevalence.

Another possible explanation to the lower risk of ALS in the first generation of immigrants could a lower detection rate, which then could be explained by a lower mental health, language and cultural differences making the detection of ALS lower. However, with a serious and life-threatening disease like ALS this seems less likely.

Regarding the incidence rate, we chose to include men and women 18 years of age and above. If adjusting to the whole population the estimated incidence rate for all ages would be 4.4 per 100,000 person-years, i.e. slightly higher than the earlier reported value of 3.8 per 100,000 person-years [7]. As regards sex distribution, we found a male to female ratio of around 1.4 in both the first- and second-generation study, i.e. close to what is found earlier [1]. The mean and median ages were around 60 years of age, i.e. what is described earlier for sporadic cases [1], although the peak incidence rate was rather high, i.e. in the age-group 75–79 years. In the first-generation study, the mean and median ages were higher than in the second-generation study, reflecting the differences in age distribution in the studied populations, although the peak incidence age-groups were similar. Besides, the mean and median ages were higher in Swedish-born men and women, as well as in men and women with Swedish-born parents, compared to the second-generation men and women, also reflecting differences in age distribution.

This study has limitations. We used the Swedish NPR, in contrast to an earlier study using data from the population-based motor neuron disease register [7]. The number of cases was quite low, why we had to categorize the individuals into a few groups, i.e., Nordic countries; Europe (except Nordic countries) and North America; and Asia, Africa and Latin America. Despite this, the number of cases were low, especially in the second-generation study among those with parents from Asia, Africa and Latin America, why these results should be interpreted with great caution. Furthermore, we did not have access to genetic data or familiarity of ALS, which would be of interest considering the differences in ALS incidence and prevalence in the world but also between different European regions which could be related to genetic differences [2, 3, 14]. The strengths lie in the Swedish registers that have been shown to be of high quality [15, 16], and we expect that we have detected most cases in the Swedish National Patient Register.

In conclusion, we found a lower ALS risk among foreign-born men and women, with lower risks for two of the groups, i.e. men and women from Europe outside the Nordic countries, and from Asia, Africa and Latin America. The lower risk among non-Western immigrants is expected, while we have no good explanation for Western immigrants. For men and women born in Sweden with foreign-born parents the risks were similar compared to native Swedes.

## Supplementary Information

Below is the link to the electronic supplementary material.

Supplementary file1 (DOCX 448 kb)

## Data Availability

The authors are not allowed to share the used data from the data sources being used.
