# Supplementary material for: Amyotrophic lateral sclerosis (ALS) among immigrant groups and Swedish-born individuals: a cohort study of all adults 18 years of age and older in Sweden
Source: J Neurol. 2021 Aug 24;269(4):1989–95. doi: 10.1007/s00415-021-10765-6 (PMC8940831; doi:10.1007/s00415-021-10765-6)
Supplement: Supplementary file 1 — Supplementary file1 (DOCX 448 kb) [file 415_2021_10765_MOESM1_ESM.docx]

**Supplementary Table 1a**

| **Population in first-generation study and number of ALS cases in men** | | | | | | | | | | | |
| --- | --- | --- | --- | --- | --- | --- | --- | --- | --- | --- | --- |
|  | Swedish born | | | | |  | Foreign born | | | | |
|  | Population | |  | Events | |  | Population | |  | Events | |
|  | No. | % |  | No | % |  | No. | % |  | No | % |
| Total population | 2450546 |  |  | 2716 |  |  | 524595 |  |  | 301 |  |
| Age (years) |  |  |  |  |  |  |  |  |  |  |  |
| 18-39 | 908502 | 37.1 |  | 176 | 6.5 |  | 266974 | 50.9 |  | 35 | 11.6 |
| 40-49 | 419065 | 17.1 |  | 377 | 13.9 |  | 95930 | 18.3 |  | 48 | 15.9 |
| 50-59 | 449414 | 18.3 |  | 799 | 29.4 |  | 83128 | 15.8 |  | 93 | 30.9 |
| 60+ | 673565 | 27.5 |  | 1364 | 50.2 |  | 78563 | 15.0 |  | 125 | 41.5 |
| Educational level |  |  |  |  |  |  |  |  |  |  |  |
| ≤ 9 | 810317 | 33.1 |  | 1089 | 40.1 |  | 315900 | 60.2 |  | 105 | 34.9 |
| 10-11 | 1064661 | 43.4 |  | 1013 | 37.3 |  | 124969 | 23.8 |  | 124 | 41.2 |
| ≥ 12 | 575568 | 23.5 |  | 614 | 22.6 |  | 83726 | 16.0 |  | 72 | 23.9 |
| Region of residence |  |  |  |  |  |  |  |  |  |  |  |
| Large cities | 1107548 | 45.2 |  | 1257 | 46.3 |  | 191677 | 36.5 |  | 166 | 55.1 |
| Southern Sweden | 830258 | 33.9 |  | 890 | 32.8 |  | 86073 | 16.4 |  | 86 | 28.6 |
| Northern Sweden | 512740 | 20.9 |  | 569 | 20.9 |  | 246845 | 47.1 |  | 49 | 16.3 |
| Marital status |  |  |  |  |  |  |  |  |  |  |  |
| Married | 1359862 | 55.5 |  | 2001 | 73.7 |  | 405462 | 77.3 |  | 224 | 74.4 |
| Not married | 1090684 | 44.5 |  | 715 | 26.3 |  | 119133 | 22.7 |  | 77 | 25.6 |
| Neighborhood deprivation |  |  |  |  |  |  |  |  |  |  |  |
| Low | 391949 | 16.0 |  | 473 | 17.4 |  | 25554 | 4.9 |  | 39 | 13.0 |
| Middle | 1243110 | 50.7 |  | 1376 | 50.7 |  | 100159 | 19.1 |  | 107 | 35.5 |
| High | 281509 | 11.5 |  | 296 | 10.9 |  | 79558 | 15.2 |  | 52 | 17.3 |
| Unknown | 533978 | 21.8 |  | 571 | 21.0 |  | 319324 | 60.9 |  | 103 | 34.2 |
| Diagnosis of diabetes |  |  |  |  |  |  |  |  |  |  |  |
| Non | 2263485 | 92.4 |  | 2496 | 91.9 |  | 490606 | 93.5 |  | 273 | 90.7 |
| Yes | 187061 | 7.6 |  | 220 | 8.1 |  | 33989 | 6.5 |  | 28 | 9.3 |
| Diagnosis of Heart diseases |  |  |  |  |  |  |  |  |  |  |  |
| Non | 1921190 | 78.4 |  | 2071 | 76.3 |  | 459902 | 87.7 |  | 233 | 77.4 |
| Yes | 529356 | 21.6 |  | 645 | 23.7 |  | 64693 | 12.3 |  | 68 | 22.6 |
| Diagnosis of dementia |  |  |  |  |  |  |  |  |  |  |  |
| Non | 2385428 | 97.3 |  | 2620 | 96.5 |  | 518578 | 98.9 |  | 290 | 96.3 |
| Yes | 65118 | 2.7 |  | 96 | 3.5 |  | 6017 | 1.1 |  | 11 | 3.7 |

**Supplementary Table 1b.**

| **Population in first-generation study and number of ALS cases in women** | | | | | | | | | | |  |
| --- | --- | --- | --- | --- | --- | --- | --- | --- | --- | --- | --- |
|  | Swedish born | | | | |  | Foreign born | | | | |
|  | Population | |  | Events | |  | Population | |  | Events | |
|  | No. | % |  | No | % |  | No. | % |  | No | % |
| Total population | 2642631 |  |  | 2084 |  |  | 510926 |  |  | 243 |  |
| Age (years) |  |  |  |  |  |  |  |  |  |  |  |
| 18-39 | 904904 | 34.2 |  | 129 | 6.2 |  | 257056 | 50.3 |  | 22 | 9.1 |
| 40-49 | 433326 | 16.4 |  | 256 | 12.3 |  | 94285 | 18.5 |  | 45 | 18.5 |
| 50-59 | 463102 | 17.5 |  | 577 | 27.7 |  | 72023 | 14.1 |  | 70 | 28.8 |
| 60+ | 841299 | 31.8 |  | 1122 | 53.8 |  | 87562 | 17.1 |  | 106 | 43.6 |
| Educational level |  |  |  |  |  |  |  |  |  |  |  |
| ≤ 9 | 868014 | 32.8 |  | 900 | 43.2 |  | 297113 | 58.2 |  | 115 | 47.3 |
| 10-11 | 802047 | 30.4 |  | 676 | 32.4 |  | 79268 | 15.5 |  | 67 | 27.6 |
| ≥ 12 | 972570 | 36.8 |  | 508 | 24.4 |  | 134545 | 26.3 |  | 61 | 25.1 |
| Region of residence |  |  |  |  |  |  |  |  |  |  |  |
| Large cities | 1210254 | 45.8 |  | 950 | 45.6 |  | 203554 | 39.8 |  | 140 | 57.6 |
| Southern Sweden | 900990 | 34.1 |  | 697 | 33.4 |  | 91663 | 17.9 |  | 61 | 25.1 |
| Northern Sweden | 531387 | 20.1 |  | 437 | 21.0 |  | 215709 | 42.2 |  | 42 | 17.3 |
| Marital status |  |  |  |  |  |  |  |  |  |  |  |
| Married | 1334897 | 50.5 |  | 1309 | 62.8 |  | 377965 | 74.0 |  | 146 | 60.1 |
| Not married | 1307734 | 49.5 |  | 775 | 37.2 |  | 132961 | 26.0 |  | 97 | 39.9 |
| Neighborhood deprivation |  |  |  |  |  |  |  |  |  |  |  |
| Low | 413913 | 15.7 |  | 351 | 16.8 |  | 29062 | 5.7 |  | 35 | 14.4 |
| Middle | 1350940 | 51.1 |  | 1025 | 49.2 |  | 114653 | 22.4 |  | 91 | 37.4 |
| High | 320120 | 12.1 |  | 283 | 13.6 |  | 79885 | 15.6 |  | 35 | 14.4 |
| Unknown | 557658 | 21.1 |  | 425 | 20.4 |  | 287326 | 56.2 |  | 82 | 33.7 |
| Diagnosis of diabetes |  |  |  |  |  |  |  |  |  |  |  |
| Non | 2484417 | 94.0 |  | 1968 | 94.4 |  | 485728 | 95.1 |  | 221 | 90.9 |
| Yes | 158214 | 6.0 |  | 116 | 5.6 |  | 25198 | 4.9 |  | 22 | 9.1 |
| Diagnosis of Heart diseases |  |  |  |  |  |  |  |  |  |  |  |
| Non | 2205279 | 83.5 |  | 1775 | 85.2 |  | 461614 | 90.3 |  | 202 | 83.1 |
| Yes | 437352 | 16.5 |  | 309 | 14.8 |  | 49312 | 9.7 |  | 41 | 16.9 |
| Diagnosis of dementia |  |  |  |  |  |  |  |  |  |  |  |
| Non | 2548611 | 96.4 |  | 2015 | 96.7 |  | 501962 | 98.2 |  | 235 | 96.7 |
| Yes | 94020 | 3.6 |  | 69 | 3.3 |  | 8964 | 1.8 |  | 8 | 3.3 |

**Supplementary Table 2a.**

| **Population in second-generation study and number of ALS cases in men** | | | | | | | | | | | |
| --- | --- | --- | --- | --- | --- | --- | --- | --- | --- | --- | --- |
|  | Swedish born parents | | | | |  | Foreign born parents | | | | |
|  | Population | |  | Events | |  | Population | |  | Events | |
|  | No. | % |  | No | % |  | No. | % |  | No | % |
| Total population | 2071939 |  |  | 1877 |  |  | 274916 |  |  | 150 |  |
| Age (years) |  |  |  |  |  |  |  |  |  |  |  |
| 18-39 | 982268 | 47.4 |  | 198 | 10.5 |  | 190687 | 69.4 |  | 40 | 26.7 |
| 40-49 | 451452 | 21.8 |  | 402 | 21.4 |  | 54966 | 20.0 |  | 50 | 33.3 |
| 50-59 | 463950 | 22.4 |  | 804 | 42.8 |  | 23467 | 8.5 |  | 46 | 30.7 |
| 60+ | 174269 | 8.4 |  | 473 | 25.2 |  | 5796 | 2.1 |  | 14 | 9.3 |
| Educational level |  |  |  |  |  |  |  |  |  |  |  |
| ≤ 9 | 549188 | 26.5 |  | 642 | 34.2 |  | 68327 | 24.9 |  | 36 | 24.0 |
| 10-11 | 971318 | 46.9 |  | 772 | 41.1 |  | 133207 | 48.5 |  | 66 | 44.0 |
| ≥ 12 | 551433 | 26.6 |  | 463 | 24.7 |  | 73382 | 26.7 |  | 48 | 32.0 |
| Region of residence |  |  |  |  |  |  |  |  |  |  |  |
| Large cities | 954328 | 46.1 |  | 870 | 46.4 |  | 156366 | 56.9 |  | 88 | 58.7 |
| Southern Sweden | 727101 | 35.1 |  | 594 | 31.6 |  | 79759 | 29.0 |  | 43 | 28.7 |
| Northern Sweden | 390510 | 18.8 |  | 413 | 22.0 |  | 38791 | 14.1 |  | 19 | 12.7 |
| Marital status |  |  |  |  |  |  |  |  |  |  |  |
| Married | 880847 | 42.5 |  | 1187 | 63.2 |  | 85881 | 31.2 |  | 74 | 49.3 |
| Not married | 1191092 | 57.5 |  | 690 | 36.8 |  | 189035 | 68.8 |  | 76 | 50.7 |
| Neighborhood deprivation |  |  |  |  |  |  |  |  |  |  |  |
| Low | 364003 | 17.6 |  | 336 | 17.9 |  | 35790 | 13.0 |  | 25 | 16.7 |
| Middle | 1163721 | 56.2 |  | 1065 | 56.7 |  | 122625 | 44.6 |  | 58 | 38.7 |
| High | 266748 | 12.9 |  | 203 | 10.8 |  | 50323 | 18.3 |  | 28 | 18.7 |
| Unknown | 277467 | 13.4 |  | 273 | 14.5 |  | 66178 | 24.1 |  | 39 | 26.0 |
| Diagnosis of diabetes |  |  |  |  |  |  |  |  |  |  |  |
| Non | 1935288 | 93.4 |  | 1724 | 91.8 |  | 262272 | 95.4 |  | 138 | 92.0 |
| Yes | 136651 | 6.6 |  | 153 | 8.2 |  | 12644 | 4.6 |  | 12 | 8.0 |
| Diagnosis of Heart diseases |  |  |  |  |  |  |  |  |  |  |  |
| Non | 1779503 | 85.9 |  | 1540 | 82.0 |  | 252164 | 91.7 |  | 122 | 81.3 |
| Yes | 292436 | 14.1 |  | 337 | 18.0 |  | 22752 | 8.3 |  | 28 | 18.7 |
| Diagnosis of dementia |  |  |  |  |  |  |  |  |  |  |  |
| Non | 2054758 | 99.2 |  | 1810 | 96.4 |  | 274045 | 99.7 |  | 146 | 97.3 |
| Yes | 17181 | 0.8 |  | 67 | 3.6 |  | 871 | 0.3 |  | 4 | 2.7 |

**Supplementary Table 2b.**

| **Population in second-generation study and number of ALS cases in women** | | | | | | | | | | | |
| --- | --- | --- | --- | --- | --- | --- | --- | --- | --- | --- | --- |
|  | Swedish born parents | | | | |  | Foreign born parents | | | | |
|  | Population | |  | Events | |  | Population | |  | Events | |
|  | No. | % |  | No | % |  | No. | % |  | No | % |
| Total population | 1984289 |  |  | 1286 |  |  | 257701 |  |  | 107 |  |
| Age (years) |  |  |  |  |  |  |  |  |  |  |  |
| 18-39 | 927898 | 46.8 |  | 130 | 10.1 |  | 177908 | 69.0 |  | 26 | 24.3 |
| 40-49 | 431391 | 21.7 |  | 252 | 19.6 |  | 51814 | 20.1 |  | 42 | 39.3 |
| 50-59 | 448222 | 22.6 |  | 578 | 44.9 |  | 22149 | 8.6 |  | 27 | 25.2 |
| 60+ | 176778 | 8.9 |  | 326 | 25.3 |  | 5830 | 2.3 |  | 12 | 11.2 |
| Educational level |  |  |  |  |  |  |  |  |  |  |  |
| ≤ 9 | 448219 | 22.6 |  | 391 | 30.4 |  | 59280 | 23.0 |  | 31 | 29.0 |
| 10-11 | 941146 | 47.4 |  | 585 | 45.5 |  | 124199 | 48.2 |  | 50 | 46.7 |
| ≥ 12 | 594924 | 30.0 |  | 310 | 24.1 |  | 74222 | 28.8 |  | 26 | 24.3 |
| Region of residence |  |  |  |  |  |  |  |  |  |  |  |
| Large cities | 931475 | 46.9 |  | 585 | 45.5 |  | 148623 | 57.7 |  | 62 | 57.9 |
| Southern Sweden | 690430 | 34.8 |  | 444 | 34.5 |  | 73587 | 28.6 |  | 27 | 25.2 |
| Northern Sweden | 362384 | 18.3 |  | 257 | 20.0 |  | 35491 | 13.8 |  | 18 | 16.8 |
| Marital status |  |  |  |  |  |  |  |  |  |  |  |
| Married | 929882 | 46.9 |  | 804 | 62.5 |  | 96034 | 37.3 |  | 58 | 54.2 |
| Not married | 1054407 | 53.1 |  | 482 | 37.5 |  | 161667 | 62.7 |  | 49 | 45.8 |
| Neighborhood deprivation |  |  |  |  |  |  |  |  |  |  |  |
| Low | 364330 | 18.4 |  | 228 | 17.7 |  | 35277 | 13.7 |  | 13 | 12.1 |
| Middle | 1111692 | 56.0 |  | 671 | 52.2 |  | 116329 | 45.1 |  | 60 | 56.1 |
| High | 247762 | 12.5 |  | 185 | 14.4 |  | 45123 | 17.5 |  | 15 | 14.0 |
| Unknown | 260505 | 13.1 |  | 202 | 15.7 |  | 60972 | 23.7 |  | 19 | 17.8 |
| Diagnosis of diabetes |  |  |  |  |  |  |  |  |  |  |  |
| Non | 1898390 | 95.7 |  | 1225 | 95.3 |  | 250229 | 97.1 |  | 103 | 96.3 |
| Yes | 85899 | 4.3 |  | 61 | 4.7 |  | 7472 | 2.9 |  | 4 | 3.7 |
| Diagnosis of Heart diseases |  |  |  |  |  |  |  |  |  |  |  |
| Non | 1829270 | 92.2 |  | 1146 | 89.1 |  | 246788 | 95.8 |  | 100 | 93.5 |
| Yes | 155019 | 7.8 |  | 140 | 10.9 |  | 10913 | 4.2 |  | 7 | 6.5 |
| Diagnosis of dementia |  |  |  |  |  |  |  |  |  |  |  |
| Non | 1967309 | 99.1 |  | 1248 | 97.0 |  | 256851 | 99.7 |  | 104 | 97.2 |
| Yes | 16980 | 0.9 |  | 38 | 3.0 |  | 850 | 0.3 |  | 3 | 2.8 |

**Supplementary Table 3**

| **Incidence of ALS in first-generation male immigrants expressed as hazard ratios (HR) with 95% confidence intervals (95% CI)** | | | | | | | | |
| --- | --- | --- | --- | --- | --- | --- | --- | --- |
|  |  | Origin Sweden | | |  | Foreign born | | |
|  |  | HR* | 95% CI | |  | HR* | 95% CI | |
| Birth year |  | **0.95** | **0.94** | **0.95** |  | **0.94** | **0.93** | **0.95** |
| Educational level (ref. ≥ 12 years) |  |  |  |  |  |  |  |  |
| ≤ 9 years |  | 1.01 | 0.92 | 1.10 |  | **1.55** | **1.16** | **2.07** |
| 10-11 years |  | 1.00 | 0.90 | 1.11 |  | **1.42** | **1.02** | **1.97** |
| Region of residence (ref. Large cities) |  |  |  |  |  |  |  |  |
| Southern Sweden |  | **0.87** | **0.80** | **0.96** |  | 0.90 | 0.68 | 1.20 |
| Northern Sweden |  | **0.88** | **0.80** | **0.97** |  | **0.39** | **0.27** | **0.57** |
| Marital status (ref. Married/cohabiting) |  | **1.24** | **1.14** | **1.36** |  | 1.03 | 0.79 | 1.34 |
| Neighborhood deprivation (ref. Low) |  |  |  |  |  |  |  |  |
| Middle |  | 0.94 | 0.85 | 1.05 |  | 0.82 | 0.57 | 1.19 |
| High |  | 0.95 | 0.82 | 1.11 |  | **0.64** | **0.42** | **0.98** |
| Unknown |  | 0.88 | 0.78 | 1.00 |  | **0.51** | **0.34** | **0.76** |
| Diagnosis of diabetes (ref. Non) |  | **0.82** | **0.71** | **0.94** |  | 0.80 | 0.54 | 1.19 |
| Diagnosis of heart diseases (ref. Non) |  | **0.57** | **0.52** | **0.63** |  | **0.70** | **0.53** | **0.94** |
| Diagnosis of dementia (ref. Non) |  | **0.68** | **0.55** | **0.83** |  | 0.95 | 0.51 | 1.75 |
| *: Full adjusted. |  |  |  |  |  |  |  |  |
|  |  |  |  |  |  |  |  |  |

**Supplementary Table 4**

| **Incidence of ALS in first-generation female immigrants expressed as hazard ratios (HR) with 95% confidence intervals (95% CI)** | | | | | | | | |
| --- | --- | --- | --- | --- | --- | --- | --- | --- |
|  |  | Origin Sweden | | |  | Foreign born | | |
|  |  | HR* | 95% CI | |  | HR* | 95% CI | |
| Birth year |  | **0.95** | **0.95** | **0.95** |  | **0.95** | **0.94** | **0.96** |
| Educational level (ref. ≥ 12 years) |  |  |  |  |  |  |  |  |
| ≤ 9 years |  | 1.08 | 0.97 | 1.20 |  | 1.19 | 0.87 | 1.64 |
| 10-11 years |  | **0.83** | **0.74** | **0.93** |  | 0.85 | 0.61 | 1.19 |
| Region of residence (ref. Large cities) |  |  |  |  |  |  |  |  |
| Southern Sweden |  | 0.94 | 0.84 | 1.04 |  | 0.72 | 0.52 | 1.00 |
| Northern Sweden |  | 1.01 | 0.89 | 1.13 |  | 0.43 | 0.30 | 0.64 |
| Marital status (ref. Married/cohabiting) |  | **1.33** | **1.22** | **1.46** |  | 0.85 | 0.65 | 1.11 |
| Neighborhood deprivation (ref. Low) |  |  |  |  |  |  |  |  |
| Middle |  | **0.87** | **0.77** | **0.99** |  | 0.68 | 0.46 | 1.00 |
| High |  | 1.05 | 0.90 | 1.24 |  | **0.47** | **0.29** | **0.75** |
| Unknown |  | 0.97 | 0.83 | 1.12 |  | **0.41** | **0.27** | **0.63** |
| Diagnosis of diabetes (ref. Non) |  | **0.73** | **0.61** | **0.89** |  | 1.11 | 0.71 | 1.74 |
| Diagnosis of heart diseases (ref. Non) |  | **0.46** | **0.40** | **0.52** |  | **0.62** | **0.43** | **0.89** |
| Diagnosis of dementia (ref. Non) |  | **0.49** | **0.38** | **0.62** |  | 0.59 | 0.29 | 1.21 |
| *: Full adjusted. |  |  |  |  |  |  |  |  |

**Supplementary Table 5**

| **Incidence of ALS in second-generation immigrants expressed as hazard ratios (HR) with 95% confidence intervals (95% CI)** | | | | | | | | |
| --- | --- | --- | --- | --- | --- | --- | --- | --- |
|  | Origin Sweden | | |  | Foreign born | | |  |
|  | HR* | 95% CI | |  | HR* | 95% CI | |  |
| Birth year | **0.92** | **0.91** | **0.92** |  | **0.91** | **0.90** | **0.92** |  |
| Gender to females (ref. Males) | **0.64** | **0.59** | **0.69** |  | **0.71** | **0.55** | **0.91** |  |
| Educational level (ref. ≥ 12 years) |  |  |  |  |  |  |  |  |
| ≤ 9 years | 1.01 | 0.93 | 1.10 |  | 0.97 | 0.72 | 1.31 |  |
| 10-11 years | 0.95 | 0.87 | 1.05 |  | 0.98 | 0.70 | 1.37 |  |
| Region of residence (ref. Large cities) |  |  |  |  |  |  |  |  |
| Southern Sweden | 0.93 | 0.86 | 1.02 |  | 0.86 | 0.63 | 1.17 |  |
| Northern Sweden | **1.16** | **1.05** | **1.28** |  | 0.83 | 0.57 | 1.21 |  |
| Marital status (ref. Married/cohabiting) | 1.04 | 0.96 | 1.12 |  | 0.95 | 0.74 | 1.24 |  |
| Neighborhood deprivation (ref. Low) |  |  |  |  |  |  |  |  |
| Middle | 1.02 | 0.92 | 1.12 |  | 1.06 | 0.73 | 1.54 |  |
| High | 1.07 | 0.94 | 1.22 |  | 1.27 | 0.81 | 1.98 |  |
| Unknown | 1.16 | 1.02 | 1.32 |  | 1.02 | 0.66 | 1.57 |  |
| Diagnosis of diabetes (ref. Non) | **0.74** | **0.65** | **0.86** |  | 0.83 | 0.50 | 1.39 |  |
| Diagnosis of heart diseases (ref. Non) | **0.61** | **0.55** | **0.68** |  | 0.81 | 0.55 | 1.18 |  |
| Diagnosis of dementia (ref. Non) | **1.55** | **1.27** | **1.88** |  | 2.09 | 0.97 | 4.49 |  |
| *: Full adjusted. |  |  |  |  |  |  |  |  |

**Supplementary Figure S1. Age-specific incidence rate (per 100 000 person years) of ALS in the first-generation individuals by gender**

**
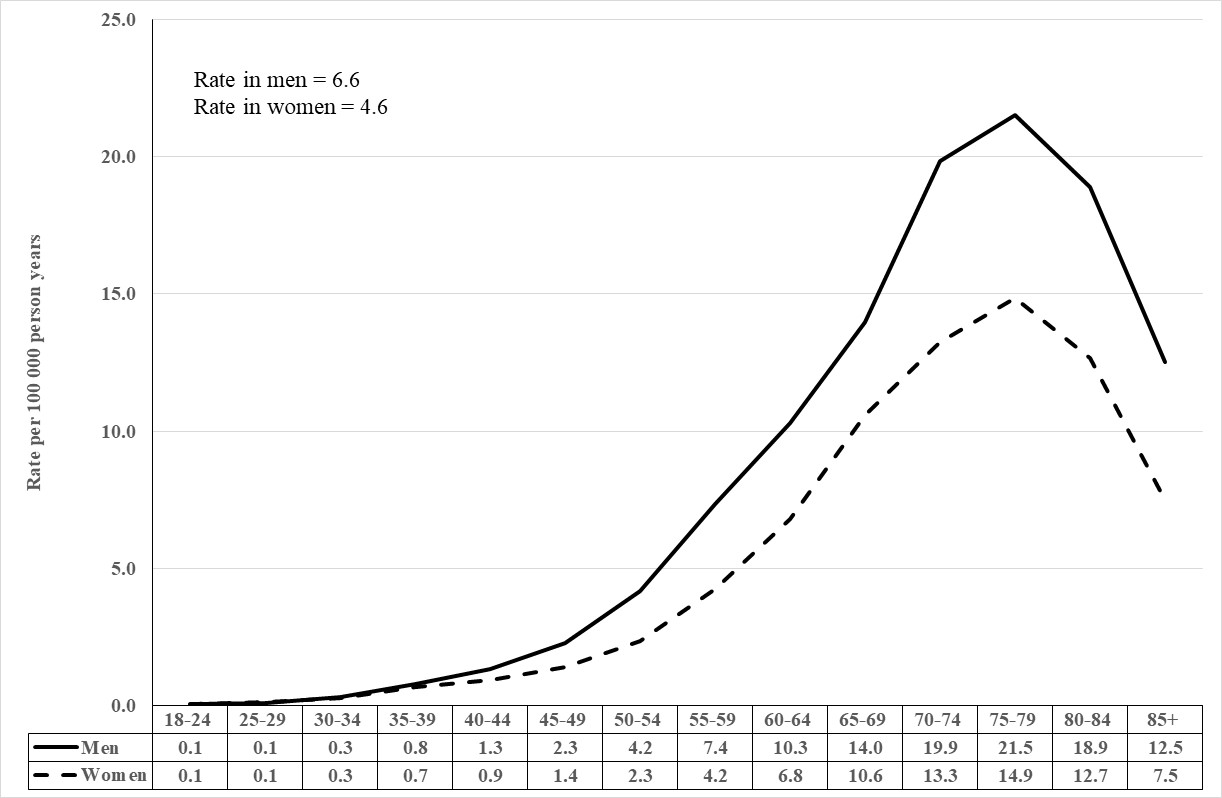
**

**Supplementary Figure S2. Age-specific incidence rate (per 100 000 person years) of ALS in the first-generation individuals by immigrant status**

**
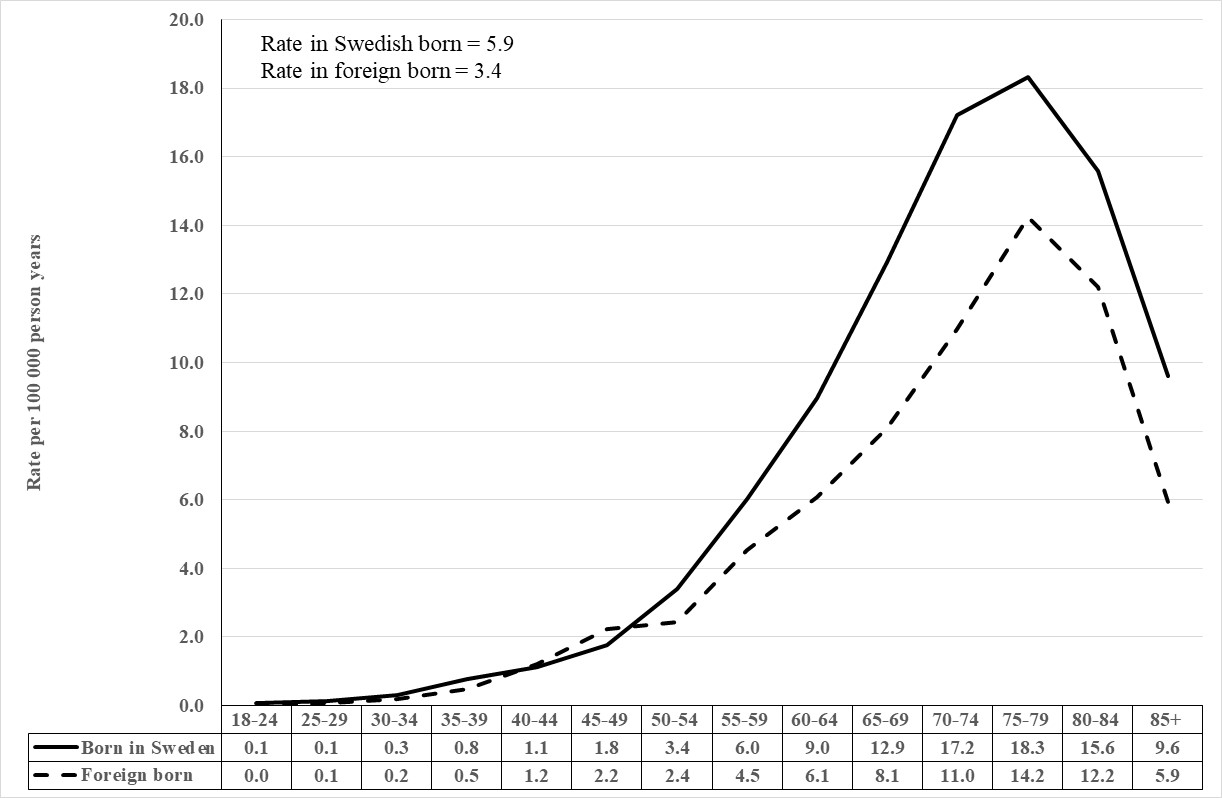
**

**Supplementary Figure S3. Age-specific incidence rate (per 100 000 person years) of ALS in the second-generation individuals by gender**

**
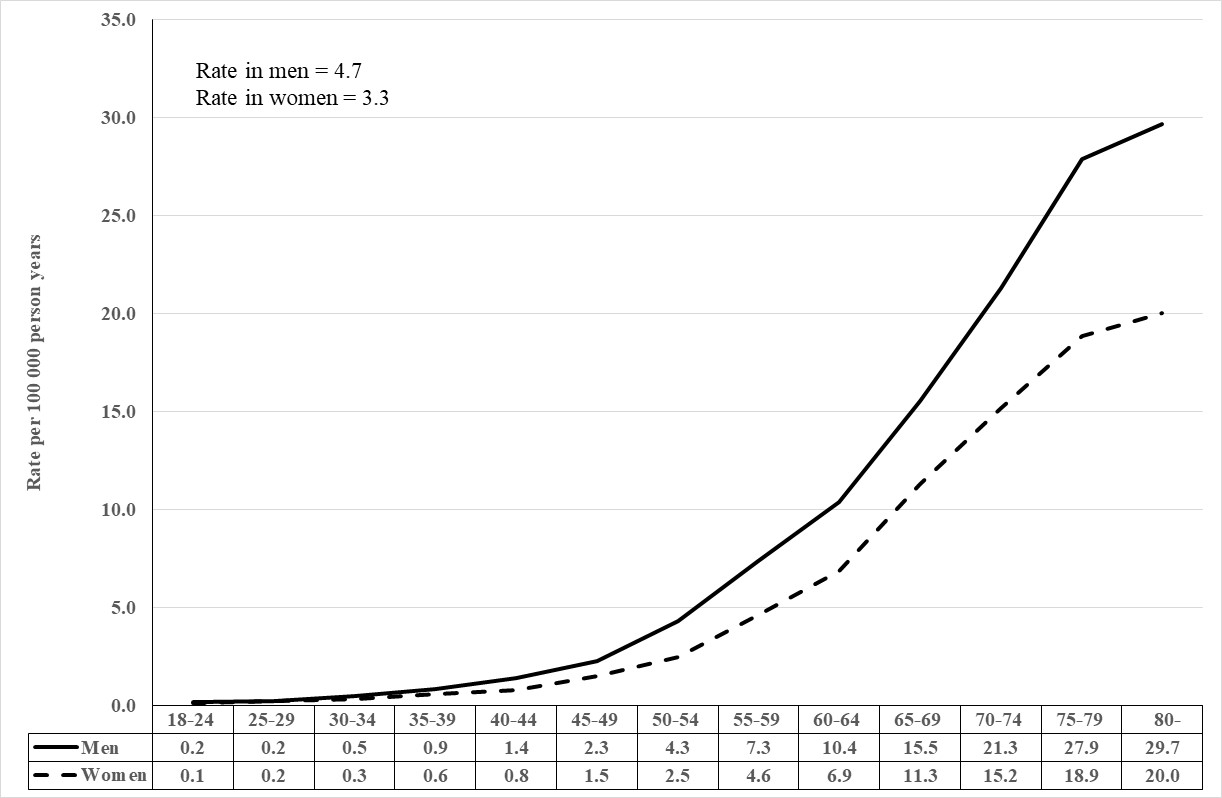
**

**Supplementary Figure S4. Age-specific incidence rate (per 100 000 person years) of ALS in the second-generation individuals by immigrant status**

**
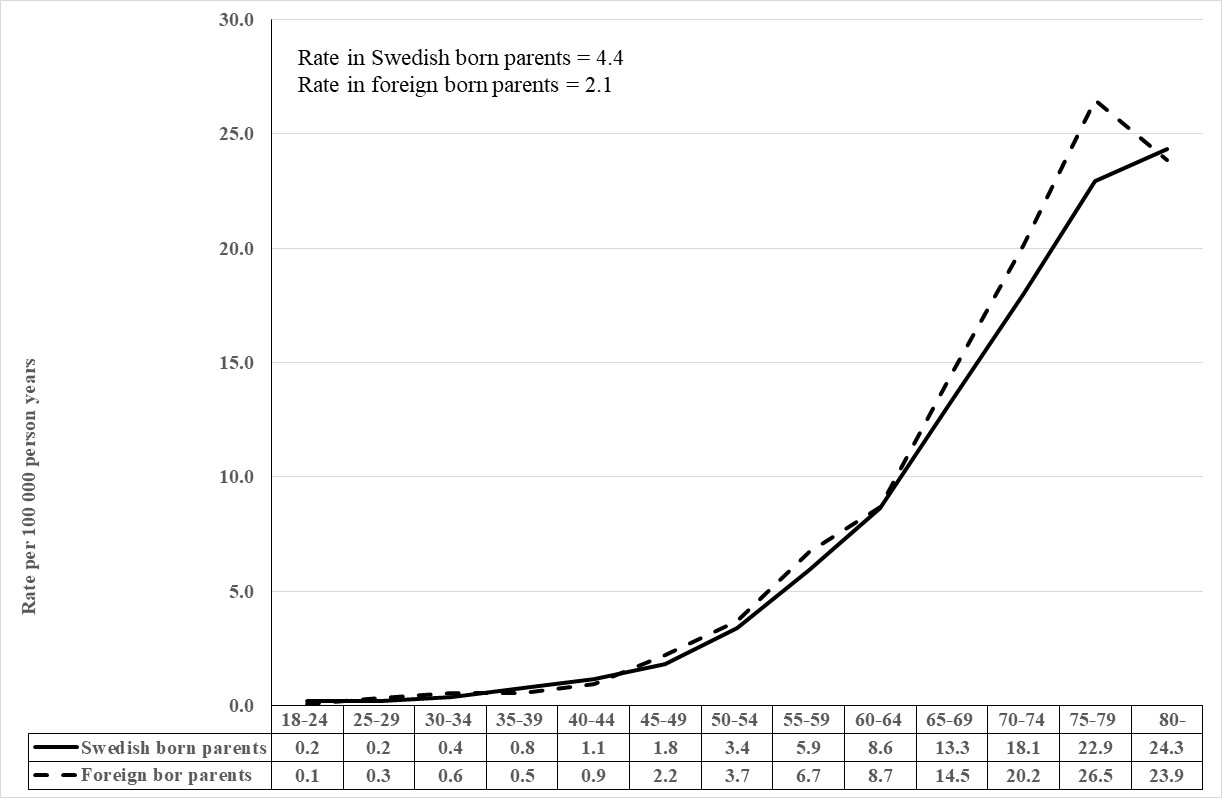
**
